# Influence of red blood cell indices on HbA1c performance in detecting dysglycaemia in a Singapore preconception cohort study

**DOI:** 10.1038/s41598-021-00445-w

**Published:** 2021-10-21

**Authors:** See Ling Loy, Jinjie Lin, Yin Bun Cheung, Aravind Venkatesh Sreedharan, Xinyi Chin, Keith M. Godfrey, Kok Hian Tan, Lynette Pei-Chi Shek, Yap Seng Chong, Melvin Khee-Shing Leow, Chin Meng Khoo, Yung Seng Lee, Shiao-Yng Chan, Ngee Lek, Jerry Kok Yen Chan, Fabian Yap

**Affiliations:** 1 Department of Reproductive Medicine, KK Women’s and Children’s Hospital, Singapore, 229899 Singapore; 2 Duke-NUS Medical School, Singapore, 169857 Singapore; 3 Singapore Institute for Clinical Sciences, Agency for Science, Technology and Research (A*STAR), Singapore, 117609 Singapore; 4 Department of Paediatrics, KK Women’s and Children’s Hospital, Singapore, 229899 Singapore; 5 Program in Health Services and Systems Research and Center for Quantitative Medicine, Duke-NUS Medical School, Singapore, 169857 Singapore; 6 Tampere Center for Child, Adolescent and Maternal Health Research, Tampere University, 33014 Tampere, Finland; 7 Medical Research Council Lifecourse Epidemiology Unit, University of Southampton, Southampton, SO16 6YD UK; 8 National Institute for Health Research Southampton Biomedical Research Centre, University of Southampton and University Hospital Southampton National Health Service Foundation Trust, Southampton, SO16 6YD UK; 9 Department of Maternal Fetal Medicine, KK Women’s and Children’s Hospital, Singapore, 229899 Singapore; 10 Department of Paediatrics, Yong Loo Lin School of Medicine, National University of Singapore, Singapore, 119228 Singapore; 11 Khoo Teck Puat-National University Children’s Medical Institute, National University Hospital, National University Health System, Singapore, 119074 Singapore; 12 Yong Loo Lin School of Medicine, National University of Singapore, National University Health System, Singapore, 119228 Singapore; 13 Cardiovascular and Metabolic Disorder Programme, Duke-NUS Medical School, Singapore, 169857 Singapore; 14 Department of Endocrinology, Tan Tock Seng Hospital, Singapore, 308433 Singapore; 15 Lee Kong Chian School of Medicine, Nanyang Technological University, Singapore, 636921 Singapore; 16 Department of Medicine, National University Hospital, Singapore, 119074 Singapore; 17 Department of Medicine, Yong Loo Lin School of Medicine, National University of Singapore, Singapore, 117597 Singapore; 18 Department of Obstetrics and Gynaecology, Yong Loo Lin School of Medicine, National University of Singapore, Singapore, 119228 Singapore

**Keywords:** Health care, Medical research

## Abstract

Abnormalities of red blood cell (RBC) indices may affect glycated haemoglobin (HbA1c) levels. We assessed the influence of haemoglobin (Hb) and mean corpuscular volume (MCV) on the performance of HbA1c in detecting dysglycaemia among reproductive aged women planning to conceive. Women aged 18–45 years (n = 985) were classified as normal (12 ≤ Hb ≤ 16 g/dL and 80 ≤ MCV ≤ 100 fL) and abnormal (Hb < 12 g/dL and/or MCV < 80 fL). The Area Under the Receiver Operating Characteristic (AUROC) curve was used to determine the performance of HbA1c in detecting dysglycaemic status (prediabetes and diabetes). There were 771 (78.3%) women with normal RBC indices. The AUROCs for the normal and abnormal groups were 0.75 (95% confidence interval 0.69, 0.81) and 0.80 (0.70, 0.90), respectively, and were not statistically different from one another [difference 0.04 (− 0.16, 0.08)]. Further stratification by ethnicity showed no difference between the two groups among Chinese and Indian women. However, Malay women with normal RBC indices displayed lower AUROC compared to those with abnormal RBC indices (0.71 (0.55, 0.87) vs. 0.98 (0.93, 1.00), p = 0.002). The results suggest that the performance of HbA1c in detecting dysglycaemia was not influenced by abnormal RBC indices based on low Hb and/or low MCV. However, there may be ethnic variations among them.

## Introduction

The 75-g oral glucose tolerance test (OGTT) is the current standard for diagnosing dysglycaemia (prediabetes and diabetes). OGTT is cumbersome because of its need for overnight fasting, intake of a glucose drink and repeated drawing of blood^1,2^. In 2009, an International Expert Committee recommended the use of HbA1c as a diagnostic alternative to OGTT^3^, leading to its strong support as a screening modality due to its lower cost, lack of need for fasting, and ability to monitor glucose control longitudinally^2,4^. While the use of HbA1c was also endorsed by the World Health Organisation (WHO) in 2010, a caveat was that the test could only be applied in the absence of conditions that may affect the accuracy of HbA1c measurements^5^. For example, anaemia has been widely reported to be a major confounder of HbA1c levels^6,7^. Shortened red blood cell (RBC) lifespan as demonstrated in haemolytic anaemia may depress HbA1c levels, whereas lengthened RBC lifespan in iron-deficiency anaemia (IDA) may elevate HbA1c levels. In addition, ethnicity has been shown to modify the diagnostic accuracy of HbA1c in diagnosing diabetes^9^.

The data on the influence of anaemia on HbA1c has mainly been derived from Western and East Asian populations, which were limited by modest sample size^2,6–8,10^. To our knowledge, the influence of anaemia on HbA1c levels among women of reproductive age in Southeast Asia is not known, despite the high prevalences of anaemia among such women in Singapore (13%), Malaysia (32%) and Indonesia (31%)^11^. In particular, those of Malay ethnicity more commonly have anaemia and haemoglobinopathy^12,13^, which may lower HbA1c and potentially lead to underdiagnosis of dysglycaemia. This would have particular implications for the assessment of women planning to conceive due to potential consequences of hyperglycaemia on subsequent pregnancy outcomes. Thus, understanding influence of anaemia on HbA1c is crucial before HbA1c could be adopted as a screening tool for detecting dysglycaemia among Southeast Asian women of reproductive age. Importantly, early detection of dysglycaemia in women planning for pregnancy will reduce the risk for birth complications^14^. Here, we examined the influence of abnormal RBC indices based on low mean corpuscular volume (MCV) and/or low haemoglobin (Hb) levels on the performance of HbA1c in detecting dysglycaemic status, defined by OGTT, among preconception women of reproductive age in Singapore. We hypothesised that the performance of HbA1c in detecting dysglycaemia among women with normal RBC indices would be greater than those with abnormal RBC indices.

## Materials and methods

### Study design and participants

Data were drawn from the Singapore PREconception Study of long-Term maternal and child Outcomes (S-PRESTO) study (clinicaltrials.gov, NCT03531658)^15^, which was conducted in accordance with the ethical principles stated in the Declaration of Helsinki. Ethical approval was granted by the SingHealth Centralised Institutional Review Board (reference 2014/692/D).

Participants were recruited from the general population between February 2015 and October 2017 and satisfied the following criteria: (a) Chinese, Malay, or Indian ethnicity, or a mixed of any two of these three ethnicities, (b) between the age of 18–45 years, and (c) planning to conceive. Participants who had been previously diagnosed with type 1 or type 2 diabetes mellitus, had taken anticonvulsant medications or oral steroids, or had received fertility treatment in the previous one month were excluded from the study. Informed written consent was obtained from all participants.

### Data collection

Detailed measurements and blood collection were conducted at the first recruitment visit, by trained research staff in the clinic of KK Women’s and Children’s Hospital, Singapore.

### MCV and Hb levels and RBC count

Overnight fasting blood samples were collected from all participants at the recruitment visits and analysed for haematological parameters. MCV, Hb and RBC count were measured as part of complete blood count using the Sysmex XE-5000 analyser (Sysmex Corporation) or XN-1000 analyser (Sysmex Corporation). Measurements by both analysers were validated in accordance to the 2017 Clinical Laboratory Standard Institutes (CLSI) guidelines.

Women were classified as normal if their Hb and MCV were 12–16 g/dL and 80–100 fL, respectively; counterparts were classified as abnormal (low Hb < 12 g/dL and/or low MCV < 100 fL)^16,17^. None of them had Hb > 16 g/dL or MCV > 100 fL. Based on Hb and MCV distributions, we further classified women with abnormal RBC indices into: (i) normal Hb and low MCV; (ii) low Hb and normal MCV; (iii) low Hb and low MCV.

### Plasma glucose concentrations and HbA1c levels

In addition to the collection of overnight fasting blood samples, all participants underwent a 75-g OGTT. To reduce glycolysis after blood collection, blood tubes containing sodium fluoride were used to collect samples for glucose testing; for HbA1c testing, samples were collected in EDTA tubes. All samples were kept cold on ice, immediately sent to lab, centrifuged within 30 min and analysed within 1 h from the time of earliest blood draw. Fasting plasma glucose (FPG), 2-h postprandial plasma glucose (2hPPG) and HbA1c levels were measured using the ARCHITECT c8000 Clinical Chemistry Analyser (Abbott Laboratories), which had received National Glycohaemoglobin Standardisation Programme certification for HbA1c testing.

Type 2 diabetes mellitus, impaired fasting glucose (IFG) and impaired glucose tolerance (IGT) were diagnosed based on: FPG ≥ 7.0 mmol/L or 2-h PG ≥ 11.1 mmol/L for type 2 diabetes mellitus; FPG 6.1 to 6.9 mmol/L and 2-h PG < 7.8 mmol/L for IFG; and FPG < 7.0 mmol/L and 2-h PG ≥ 7.8 and < 11.1 mmol/L for IGT^18^. Owing to the small number of women diagnosed with type 2 diabetes mellitus (n = 20, 2.0%) and IFG (n = 1, 0.1%), we grouped women with type 2 diabetes mellitus, IFG and/or IGT as having dysglycaemia.

### Statistical analysis

Demographic characteristics and measurements of participants with normal and abnormal RBC indices were compared using independent sample *t* test for continuous variables or Fisher’s exact test for categorical variables.

The performances of HbA1c in detecting dysglycaemia in both groups of participants were examined through receiver-operating-characteristic (ROC) curve analysis. The area-under-ROC curves (AUROC) were determined and compared for equality using methods developed by DeLong et al.^19^. The 95% confidence interval (CI) for the difference in AUROC between groups was estimated by bootstrapping, with 10,000 replicates. We defined an AUROC value of 0.90 or above as being optimal.

In view of the ethnic-dependent differences in the diagnostic accuracy of HbA1c shown in previous studies^9^, we extended our investigation to examine the performance of HbA1c in each ethnicity. ROC curves specific to Chinese, Malay, and Indian participants from each group were plotted and tested for equality. Participants of mixed-ethnicity (n = 33, 3.4%) were excluded from this analysis due to its small sample size.

To better understand the abnormalities in the RBC indices, participants with normal and abnormal RBC indices within each ethnic group were compared according to their haematological parameters, using independent sample *t* tests. The Mentzer Index was derived to differentiate women with abnormal RBC indices into those with either beta thalassaemia or IDA^20^. The Mentzer Index was computed by dividing MCV (in fL) by RBC count (× 10^6^ µL^−1^); values of < 13, > 13 and = 13 were defined as suggestive of beta thalassaemia, IDA and inconclusive, respectively. All statistical analyses were conducted using SPSS (Version 20; IBM), except for ROC analyses, which were performed using STATA (Version 16; STATA).

### Details of ethics approval

The Singhealth Centralised Institute Review Board approved the study protocol (reference 2014/692/D). All participants provided written informed consent. This study was registered at www.clinicaltrials.gov, NCT03531658 (22/05/2018).

## Results

Of the 1032 eligible women enrolled, 985 (95.4%) women with complete dataset were included for analysis. As shown in Table 1, 771 (78.3%) and 214 (21.7%) had normal and abnormal RBC indices, respectively. Among those with abnormal RBC indices, 45.8% (n = 98) had both low Hb and low MCV, 28.5% (n = 61) had low MCV only, 25.7% (n = 55) had low Hb only. Compared to women with normal RBC indices, those with abnormal RBC indices were more likely to be Malay and Indian ethnicities, and had higher 2hPPG. There were no differences between the groups of women with normal and abnormal RBC indices in relation to age, HbA1c, FPG and dysglycaemic status.

**Table 1:** Characteristics of women with normal and abnormal RBC indices from the S-PRESTO study.

	All	Normal RBC indices	Abnormal RBC indices		Normal Hb and low MCV	Low Hb and normal MCV	Low Hb and low MCV	
	(n = 985)	(n = 771)	(n = 214)	p*	(n = 61)	(n = 55)	(n = 98)	p*
	Mean ± SD or n (%)				mean ± SD or n (%)			
Age, years	30.77 ± 3.74	30.70 ± 3.76	31.05 ± 3.68	0.228	29.90 ± 3.56	31.55 ± 3.61	31.48 ± 3.67	0.024
**Ethnicity**								
Chinese	711 (72.2)	589 (76.4)	122 (57.0)	< 0.001	24 (39.3)	38 (69.1)	60 (61.2)	< 0.001
Malay	152 (15.4)	104 (13.5)	48 (22.4)		14 (23.0)	9 (16.4)	25 (25.5)	
Indian	89 (9.0)	57 (7.4)	32 (15.0)		16 (26.2)	7 (12.7)	9 (9.2)	
Mixed	33 (3.4)	21 (2.7)	12 (5.6)		7 (11.5)	1 (1.8)	4 (4.1)	
**Hb, g/dL**	12.92 ± 1.18	13.33 ± 0.75	11.46 ± 1.25	< 0.001	12.76 ± 0.70	11.42 ± 0.47	10.66 ± 1.13	< 0.001
Normal ≥ 12 g/dL	832 (84.5)	771 (100.0)	61 (28.5)	< 0.001	61 (100.0)	0	0	< 0.001
Low < 12 g/dL	153 (15.5)	0	153 (71.5)		0	55 (100.0)	98 (100.0)	
**MCV, fL**	84.92 ± 7.17	87.62 ± 3.53	75.20 ± 8.45	< 0.001	74.78 ± 5.32	85.72 ± 3.58	69.54 ± 6.09	< 0.001
Normal 80–100 fL	826 (83.9)	771 (100.0)	55 (25.7)	< 0.001	0	55 (100.0)	0	< 0.001
Low < 80 fL	159 (16.1)	0	159 (74.3)		61 (100.0)	0	98 (100.0)	
HbA1c, %	5.14 ± 0.46	5.13 ± 0.48	5.17 ± 0.40	0.277	5.28 ± 0.59	5.09 ± 0.26	5.14 ± 0.28	0.069
Fasting plasma glucose, mmol/L	4.82 ± 0.74	4.81 ± 0.74	4.87 ± 0.71	0.339	5.03 ± 1.17	4.67 ± 0.34	4.87 ± 0.40	0.050
2 h post-load plasma glucose, mmol/L	6.01 ± 2.07	5.94 ± 2.02	6.26 ± 2.24	0.041	6.80 ± 3.37	5.95 ± 1.24	6.11 ± 1.68	0.019
**Dysglycaemia**								
No	877 (89.0)	688 (89.2)	189 (88.3)	0.711	49 (80.3)	51 (92.7)	89 (90.8)	0.151
Yes	108 (11.0)	83 (10.8)	25 (11.7)		12 (19.7)	4 (7.3)	9 (9.2)	

*Hb* haemoglobin, *HbA1c* glycated haemoglobin, *MCV* mean corpuscular volume, *RBC* red blood cells, *SD* standard deviation, *S-PRESTO* Singapore PREconception Study of long-Term maternal and child Outcomes.

*By independent sample *t* test or one-way ANOVA for continuous variable, and Fisher’s exact test for categorical variable.

**Figure 1:**
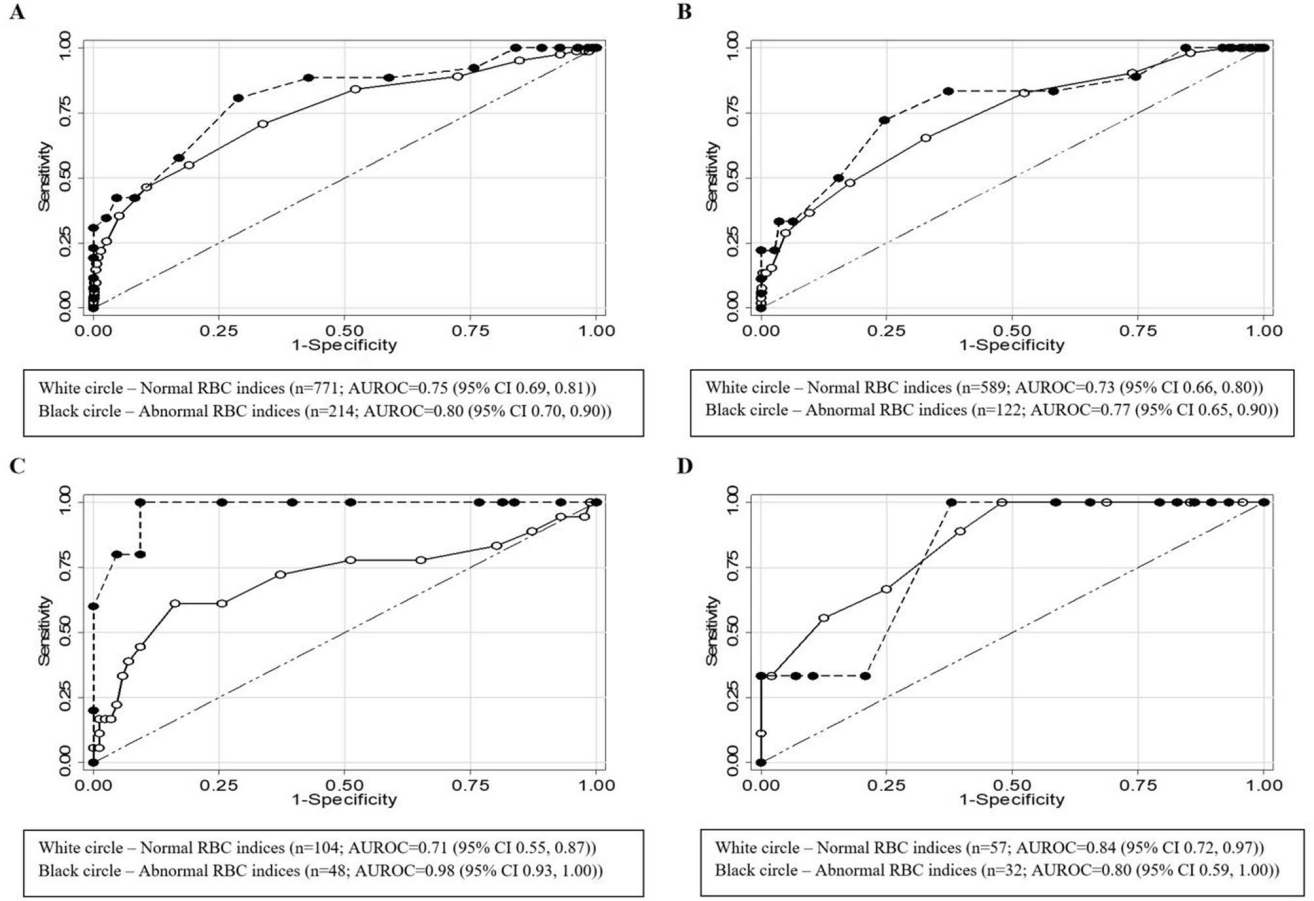
ROC curves for HbA1c in detecting dysglycaemia among women of (**A**) all ethnicities, (**B**) Chinese ethnicity, (**C**) Malay ethnicity, and (**D**) Indian ethnicity, from the S-PRESTO study. The dotted diagonal line represents AUROC of 0.5, which suggests no discrimination. *AUROC* area-under-receiver-operating-characteristic curve, *CI* confidence interval, *HbA1c* glycated haemoglobin, *RBC* red blood cell, *ROC curve* receiver-operating-characteristics curve, *S-PRESTO* Singapore PREconception Study of long-Term maternal and child Outcomes.

The ROC curves for HbA1c in detecting dysglycaemia among women with normal and abnormal RBC indices are shown in Fig. 1. The sensitivities and specificities of HbA1c at different cut-off values are shown in Supplementary Table S1 online. The AUROC for participants with normal and abnormal RBC indices were both modest, 0.75 (95% CI 0.69, 0.81) and 0.80 (0.70, 0.90), respectively, and were not statistically different from one another [difference 0.04 (− 0.16, 0.08)] (Fig. 1A). In both Chinese (Fig. 1B) and Indian (Fig. 1D), AUROC of women with normal RBC indices were not statistically different from those with abnormal RBC indices (Chinese, 0.73 (0.66, 0.80) vs. 0.77 (0.65, 0.90), p = 0.550; Indian, 0.84 (0.72, 0.97) vs. 0.80 (0.59, 1.00), p = 0.770). However, in the Malay group (Fig. 1C), AUROC of women with normal RBC indices was statistically lower than that of those with abnormal RBC indices (0.71 (0.55, 0.87) vs. 0.98 (0.93, 1.00), p = 0.002). Among women with normal RBC indices, the AUROC of the different ethnicities ranged from 0.71 to 0.84, which were not substantially different from their pooled estimate of 0.75.

Table 2 shows the haematological parameters of women with normal and abnormal RBC indices with stratification by ethnic groups. Among Chinese and Indian participants, RBC count was higher in women with abnormal RBC indices, whereas among Malay participants, no difference was observed. Based on the Mentzer Index, in all ethnicities, the majority of the women had abnormal RBC indices suggestive of IDA rather than beta thalassaemia.

**Table 2:** Haematological parameters of women from S-PRESTO study with normal and abnormal RBC indices, stratified by ethnic group.

	Normal RBC indices		Abnormal RBC indices		p*
	n	mean ± SD/%	n	mean ± SD/%	
**Chinese**					
Dysglycaemia					0.131
No	536	91.0	105	86.1	
Yes	53	9.0	17	13.9	
HbA1c, %	589	5.11 ± 0.46	122	5.14 ± 0.32	0.516
HbA1c, mmol/mol	589	32.21 ± 5.06	122	32.57 ± 3.56	0.461
Hb, g/dL	589	13.33 ± 0.77	122	11.28 ± 1.20	< 0.001
MCV, fL	589	88.17 ± 3.36	122	74.80 ± 9.79	< 0.001
RBC count, × 10^6^ µL^−1^	589	4.54 ± 0.29	122	4.80 ± 0.74	< 0.001
Mentzer Index	589	19.54 ± 1.77	122	16.20 ± 4.39	< 0.001
Beta thalassaemia	–	–	37	30.3	
IDA	–	–	85	69.7	
**Malay**					
Dysglycaemia					0.336
No	86	82.7	43	89.6	
Yes	18	17.3	5	10.4	
HbA1c, %	104	5.20 ± 0.63	48	5.17 ± 0.40	0.799
HbA1c, mmol/mol	104	33.27 ± 6.88	48	32.94 ± 4.38	0.760
Hb, g/dL	104	13.39 ± 0.70	48	11.46 ± 1.32	< 0.001
MCV, fL	104	86.01 ± 3.47	48	75.43 ± 6.49	< 0.001
RBC count, × 10^6^ µL^−1^	104	4.70 ± 0.28	48	4.77 ± 0.49	0.375
Mentzer Index	104	18.39 ± 1.63	48	16.05 ± 2.66	< 0.001
Beta thalassaemia	–	–	5	10.4	
IDA	–	–	43	89.6	
**Indian**					
Dysglycaemia					0.525
No	48	84.2	29	90.6	
Yes	9	15.8	3	9.4	
HbA1c, %	57	5.15 ± 0.30	32	5.24 ± 0.63	0.365
HbA1c, mmol/mol	57	32.7 ± 3.33	32	33.69 ± 6.82	0.362
Hb, g/dL	57	13.12 ± 0.72	32	11.94 ± 1.15	< 0.001
MCV, fL	57	85.14 ± 2.97	32	77.06 ± 5.56	< 0.001
RBC count, × 10^6^ µL^−1^	57	4.65 ± 0.31	32	4.81 ± 0.45	0.092
Mentzer Index	57	18.4 ± 1.69	32	16.22 ± 2.37	< 0.001
Beta thalassaemia	–	–	3	9.4	
IDA	–	–	29	90.6	

Values are presented as mean ± SD or n (%). *Hb* haemoglobin, *HbA1c* glycated haemoglobin, *IDA* iron deficiency anaemia, *MCV* mean corpuscular volume, *RBC* red blood cell, *S-PRESTO* Singapore PREconception Study of long-Term maternal and child Outcomes.

*By independent sample *t* test or Fisher’s exact test, as appropriate.

## Discussion

In this multi-ethnic Southeast Asian study involving reproductive-age women planning to conceive in Singapore, HbA1c displayed modest performance in detecting dysglycaemic status as indicated by AUROC < 0.90, which was not influenced by RBC indices based on abnormal MCV and/or Hb values. Upon further examining its performance by ethnicity, we noted that there were no differences in AUROC values between women of normal and abnormal RBC indices among Chinese and Indian. However, Malay women of abnormal RBC indices had greater AUROC values than their normal counterparts (0.98 vs. 0.71). These findings suggest that there may be ethnic variations for the performance of HbA1c in detecting dysglycaemia.

Modest performance of HbA1c in detecting dysglycaemia had also been reported by several Asian studies^7,21^. For example, in a Chinese study conducted by Zhou et al.^21^, the AUROC of HbA1c in detecting newly-diagnosed diabetes or prediabetes among women was 0.55. Likewise, Hardikar et al.^7^ showed that the AUROC of HbA1c for diabetes or prediabetes among Indians was 0.74. Comparatively, while the AUROC obtained in this study was slightly higher in value, it was nonetheless below the predefined optimal value of 0.90, which suggests its limitation as a diagnostic modality. Despite so, in another local study, the AUROC of HbA1c in detecting prediabetes among women was noted to be 0.91^22^. Such difference might be due to the different target groups involved. While this study included women who were incidentally found to have diabetes or prediabetes, the aforementioned study included only those with known diabetes. Although AUROC is useful to summarise overall test performance over all possible thresholds, AUROC itself cannot account for prevalence and different misclassification costs arising from false-negative and false-positive diagnoses^23^.

Anaemia are known to result in spurious elevation or reduction of HbA1c levels, which in turn altered the performance of HbA1c in other studies^2,24^. For example, in a South Korean population, IDA increases the false positive rates of HbA1c in diagnosing diabetes^2^. Others have shown that the prevalence rate of prediabetes and diabetes diagnosed by HbA1c differ between women with iron deficiency or non-iron deficiency anaemia, compared to their healthy counterparts^24^. Furthermore, depending on the measurement methods, Hb variants in haemoglobinopathies may also interfere with HbA1c measurement^25^. In this study, generally, abnormalities in RBC indices as indicated by low values of MCV and/or Hb, did not influence the performance of HbA1c in detecting dysglycaemia though there may be ethnic variations among them.

The difference in the AUROC values between those with normal and abnormal RBC indices among Malay ethnicity could be due to several reasons. It is known that Malays have a high prevalence of anaemia and haemoglobinopathy, such as Hb E-beta thalassaemia (Hb E/β-thalassaemia), which is in turn associated with higher foetal Hb (HbF) levels and may spuriously lower HbA1c value^12^. This is supported by the National Thalassaemia Registry (2011) in Singapore, which reported that 74% of registered subjects with Hb E/β-thalassaemia were of Malay ethnicity^13^. A previous local study had also shown that Malays had the highest total carrier frequency for alpha- and beta-thalassaemia mutations compared to Chinese and Indians^26^. Consequently, the HbA1c levels among Malay women with abnormal RBC indices might have been altered such that it became considerably lower than that of its reference standard, made up of Malay women with normal RBC indices. We speculate that the potential implication of such lowered HbA1c may lead to underdiagnosis of dysglycaemia among the group. Such ethnic-specific difference is consistent with several studies on Western populations. For example, in both the presence and absence of diabetes, blacks had shown higher HbA1c levels than whites, suggesting that HbA1c, if used, might lead to an overdiagnosis of dysglycaemia among the former^27,28^.

In support of our speculations, the HbA1c of Malay women with abnormal RBC indices was lower than their normal counterparts, albeit not significantly so. Based on the Mentzer Index, only a minority of them were likely to have beta thalassaemia. As the Mentzer Index only serves as a discrimination index rather than giving a definitive diagnosis of thalassaemia or IDA, the classification of women may hence be inaccurate and represents a major limitation of our study. To better clarify the suggestion that the performance of HbA1c differs by RBC indices among Malay women, further investigations taking account of the women’s thalassaemia status, total body iron and type of RBC abnormality are required. Importantly, this should be addressed in a larger ethnic-specific study, specially designed and powered to address this topic. Nonetheless, the unexpectedly better performance of HbA1c in detecting dysglycaemia among Malay participants with abnormal RBC indices might reflect a limitation of the OGTT, when a standard dextrose challenge is used to measure the glycaemic status for such women who had higher body mass index (BMI) and/or subcutaneous fat mass^29–32^.

There are several limitations to the present study. Given that this study was restricted to planned pregnancies among three Asian ethnicities in Singapore, generalizability of findings is potentially limited. Despite study recruitment from both hospital and community settings, women planning to conceive and willing to participate in this study were expected to be more health conscious and have better health status than the general population, which could introduce selection bias. Thus, it is reasonable to speculate that among women with abnormal RBC indices, a majority might be only minimally or transiently iron deficient, which could have contributed to the undetectable difference in HbA1c between women with normal and abnormal RBC indices at baseline, as well as the overall non-influential effect of abnormal RBC indices on dysglycaemic status. It is noteworthy that dysglycaemic status as defined during a single OGTT might misclassify glycaemic status, contributing to some of the discordance between HbA1c and OGTT. Further follow-up study to evaluate risks for complications of dysglycaemia is a possible approach to confirm the presence of abnormal glucose tolerance. Otherwise, future studies should consider repeated and different glucose loading according to body size for improved OGTT accuracy in defining dysglycaemic status.

## Conclusion

Among women of reproductive age in Singapore, dysglycaemic status as defined by the OGTT, was not influenced by abnormal RBC indices based on low Hb and/or low MCV. However, ethnicity might influence the performance of HbA1c in detecting dysglycaemia due to the presence of abnormal MCV and/or Hb levels. Larger study is warranted to confirm the findings and to examine reasons underlying the ethnic differences in the performance of HbA1c for detecting dysglycaemia. This is crucial in the effort to maximize the role of HbA1c by establishing ethnic-specific HbA1c cut-offs for early identification of dysglycaemia in particular populations.

## Supplementary Information

Supplementary Table S1.

## Data Availability

The data that support the findings of this study are available from S-PRESTO. Restrictions apply to the availability of these data, which were used under license for this study. Data are available from the authors with the permission of S-PRESTO.
