# Supplementary material for: Influence of red blood cell indices on HbA1c performance in detecting dysglycaemia in a Singapore preconception cohort study
Source: Sci Rep. 2021 Oct 21;11:20850. doi: 10.1038/s41598-021-00445-w (PMC8531017; doi:10.1038/s41598-021-00445-w)
Supplement: Supplementary file 1 — Supplementary Table S1. [file 41598_2021_445_MOESM1_ESM.pdf]

Influence of red blood cell indices on HbA1c performance in detecting dysglycaemia in a Singapore preconception cohort study

See Ling Loy, Jinjie Lin, Yin Bun Cheung, Aravind Venkatesh Sreedharan, Xinyi Chin, Keith M. Godfrey, Kok Hian Tan, Lynette Pei-Chi Shek, Yap Seng Chong, Melvin Khee-Shing Leow, Chin Meng Khoo, Yung Seng Lee, Shiao-Yng Chan, Ngee Lek, Jerry Kok Yen Chan, Fabian Yap*

Supplementary Table S1 Sensitivities and specificities of HbA1c in detecting dysglycaemia in women with normal and abnormal RBC indices

	Normal RBC indices		Abnormal RBC indices	
HbA1c (%)	Sensitivity	Specificity	Sensitivity	Specificity
4.6	0.99	0.02	1.00	0.04
4.7	0.99	0.04	1.00	0.07
4.8	0.98	0.07	1.00	0.11
4.9	0.95	0.15	1.00	0.16
5.0	0.89	0.28	0.92	0.23
5.1	0.84	0.48	0.88	0.41
5.2	0.71	0.66	0.88	0.57
5.3	0.55	0.81	0.80	0.70
5.4	0.47	0.90	0.56	0.83
5.5	0.36	0.95	0.40	0.92
5.6	0.27	0.97	0.40	0.95
5.7	0.23	0.99	0.32	0.97
5.8	0.20	0.99	0.28	1.00
5.9	0.17	0.99	0.24	1.00
6.0	0.14	1.00	0.22	1.00
6.1	0.10	1.00	0.20	1.00

HbA1c, glycated hemoglobin; RBC, red blood cell.
